# Medical Intelligence

**Published:** 1849

**Authors:** 


### ARTICLE X.

### MISCELLANEOUS MEDICAL INTELLIGENCE.

Antidote to Strychnine.—It is said that camphor is an effectual antidote to strychnine. Five grains given in almond emulsion stopped the tetanic symptoms almost immediately in a case where one eighth of a grain had been taken.

The next meeting of the National Medical Association will be held in Boston commencing on the first Tuesday in May next. Societies are requested to send a list of their delegates to Dr. H. J. Bowditch, Secr'y, by the 14th of April. Each society is entitled to one delegate for every ten members, and one for every additional fraction over half of that number.

We see the project of establishing a National Medical College is discussed by our friend of the Buffalo Medical Journal.

We observe that a controversy has taken place between Professor Bartlett and the Editor of the Western Lancet, on account of strictures of the latter on the "Medical Philosophy" of the former.

The cholera is prevailing in Europe according to our last reports. It has almost, if not entirely subsided in this country. It is to be feared that it will break out again when warm weather comes.

Professor Williams, the distinguished Pathologist, of London, retires from the University College at the close of the present session.

Prof. T. D. Mitchell, of Lexington, Ky., has resigned his chair in Transylvania University, and accepted that of Theory and Practice in the Philadelphia College of Medicine.

The class in the Medical College of Ohio, the past session, numbered 175 students.

The Geneva Medical College has conferred the degree of M. D. upon Miss Elizabeth Blackwell, who had complied with all the requisitions of the institution. Her thesis published in the Buffalo Med. Jour., is a sensible document.

Obituary.—Dr. Prichard, author of the work on insanity, died in London, on the 22d of last December, aged 63.

Mr. Samuel Cooper, the celebrated surgeon, author of the Dictionary, died Dec. 8, 1848, aged 68. E.

